# A chromosome-level genome assembly of the Chinese cork oak (*Quercus variabilis*)

**DOI:** 10.3389/fpls.2022.1001583

**Published:** 2022-09-23

**Authors:** Biao Han, Longxin Wang, Yang Xian, Xiao-Man Xie, Wen-Qing Li, Ye Zhao, Ren-Gang Zhang, Xiaochun Qin, De-Zhu Li, Kai-Hua Jia

**Affiliations:** ^1^Key Laboratory of State Forestry and Grassland Administration Conservation and Utilization of Warm Temperate Zone Forest and Grass Germplasm Resources, Shandong Provincial Center of Forest and Grass Germplasm Resources, Jinan, China; ^2^School of Biological Science and Technology, University of Jinan, Jinan, China; ^3^Key Laboratory of Genetics and Breeding in Forest Trees and Ornamental Plants, National Engineering Laboratory for Tree Breeding, Beijing Advanced Innovation Center for Tree Breeding by Molecular Design, Ministry of Education, College of Biological Sciences and Technology, Beijing Forestry University, Beijing, China; ^4^Department of Bioinformatics, Ori (Shandong) Gene Science and Technology Co., Ltd., Weifang, China; ^5^Germplasm Bank of Wild Species, Kunming Institute of Botany, Chinese Academy of Sciences, Kunming, Yunnan, China; ^6^Key Laboratory of Crop Genetic Improvement and Ecology and Physiology, Institute of Crop Germplasm Resources, Shandong Academy of Agricultural Sciences, Jinan, China

**Keywords:** *Quercus variabilis*, genome assembly, PacBio HiFi sequencing, Hi-C sequencing, comparative genomics

## Abstract

*Quercus variabilis* (Fagaceae) is an ecologically and economically important deciduous broadleaved tree species native to and widespread in East Asia. It is a valuable woody species and an indicator of local forest health, and occupies a dominant position in forest ecosystems in East Asia. However, genomic resources from *Q. variabilis* are still lacking. Here, we present a high-quality *Q. variabilis* genome generated by PacBio HiFi and Hi-C sequencing. The assembled genome size is 787 Mb, with a contig N50 of 26.04 Mb and scaffold N50 of 64.86 Mb, comprising 12 pseudo-chromosomes. The repetitive sequences constitute 67.6% of the genome, of which the majority are long terminal repeats, accounting for 46.62% of the genome. We used *ab initio*, RNA sequence-based and homology-based predictions to identify protein-coding genes. A total of 32,466 protein-coding genes were identified, of which 95.11% could be functionally annotated. Evolutionary analysis showed that *Q. variabilis* was more closely related to *Q. suber* than to *Q. lobata* or *Q. robur.* We found no evidence for species-specific whole genome duplications in *Quercus* after the species had diverged. This study provides the first genome assembly and the first gene annotation data for *Q. variabilis.* These resources will inform the design of further breeding strategies, and will be valuable in the study of genome editing and comparative genomics in oak species.

## Introduction

*Quercus* L. (oak) is an ecologically and economically important genus of deciduous and evergreen forest ecosystems throughout the Northern Hemisphere. The genus comprises approximately 450 species (Cavender-Bares, 2016, 2019; Plomion et al., 2016), and *Quercus* species not only play pivotal roles in ecosystem functioning (e.g., biodiversity maintenance, water and soil conservation and carbon sequestration), but also provide raw materials for timber, starch, tannin, cork and medicinal resources. Due to the economic value of these trees, their presence in many common habitats, and their dominant positions in many ecosystems and landscapes across the Northern Hemisphere (Chai et al., 2016), *Quercus* species have been the focus of many genetic, ecological and evolutionary studies (Eaton et al., 2015; Gugger et al., 2021; Fu et al., 2022). However, classification of oak trees is challenging, because of the large inter-and intraspecific morphological variation, and because of the conflicting phylogenies derived from analysis of plastid and low-copy nuclear markers (Manos et al., 1999; Simeone et al., 2013, 2016; Hubert et al., 2014; Vitelli et al., 2017; Zhang et al., 2020). With the accumulation of molecular and morphological evidence, eight *Quercus* sections, corresponding to clades, have been accepted: the Old World sections *Cyclobalanopsis*, *Cerris* and *Ilex*, and the New World sections *Quercus*, *Lobatae*, *Virentes*, *Protobalanus* and *Ponticae* (Gil-Pelegrín et al., 2017; Hipp et al., 2020).

The Chinese cork oak, *Quercus variabilis* (*Q. variabilis*) belongs to the East Asian *Cerris* lineage in subgenus *Cerris* (Hipp et al., 2020). It is an important tree species in warm-temperate deciduous broadleaved woodland, and it is native to and widespread in East Asia, including China, the Korean Peninsula, Japan, Laos and Thailand (Fujiwara and Harada, 2015). *Q. variabilis* is characterized by its thick corky bark, which is peeled to make the corks used as bottle stoppers in the wine industry (Pereira, 2011), and *Q. variabilis* is also a valuable timber species. Furthermore, *Q. variabilis,* together two other East Asian oak species (*Q. acutissima* and *Q. chenii*), is proposed as an indicator species for local forest health, due to its importance in the local ecology (Zilliox and Gosselin, 2014; Chen et al., 2020b; Asbeck et al., 2021).

Previous studies investigating *Q. variabilis* have mainly focused on its morphological characteristics (Du et al., 2021; Sun et al., 2021), its responses and adaptations to climate change (Gao et al., 2020; Xia et al., 2022), or its adaptive evolution and introgression, as assessed using whole genome resequencing (Fu et al., 2022). However, to date, no nuclear genomic resources are available for *Q. variabilis*. Here, we present the first chromosome-scale high-quality genome assembly of *Q. variabilis*, generated using a combination of Pacific Biosciences high-fidelity (PacBio HiFi) and Hi-C technologies. We performed structural gene annotation, identified repetitive sequences, and also conducted a comparative genomics study with the genomes of a further 13 plants. This study will provide important resources for the further investigation of genetic diversity in *Q. variabilis* and will improve the resolution of the oak phylogeny.

## Materials and methods

### Plant materials

*Quercus variabilis* samples were collected from an ancient tree (more than 400 years old) growing in Culai Mountain National Forest Park, Shandong Province, China.

### Genomic DNA extraction and sequencing

Fresh leaves were collected and immediately frozen in liquid nitrogen for transport back to the lab. The genomic DNA was then isolated using a Plant DNeasy Mini kit (Qiagen China, Shanghai, China) according to the manufacturer’s instructions. The quality and quantity of the DNA were determined using agarose gel electrophoresis and with a NanoDrop 2000 spectrophotometer (Thermo Fisher Scientific, Waltham, MA, United States). A library of short-insert-size genomic DNA fragments of length 300–400 bp was constructed according to the manufacturer’s instructions, and was sequenced on a DNBSEQ platform (Beijing Genomics Institute, Shenzhen, China) for 150 bp pair end sequencing. For long-read sequencing, a 20 kb high-fidelity (HiFi) library was constructed following the manufacturer’s protocol^1^ on the PacBio Sequel II platform (Pacific Biosciences of California, Inc.). To increase continuity of the genome, a Hi-C library was constructed and sequenced on the DNBSEQ platform (BGI, Shenzhen, China).

### RNA extraction and sequencing

For RNA sequencing, fresh leaves, young twigs, fruits and seeds were sampled and immediately frozen in liquid nitrogen. RNA was extracted using TRIzol reagent (Invitrogen), the genomic DNA was eliminated using DNase and the samples were then mixed for RNA sequencing. We used agarose gel electrophoresis, a NanoDrop 2000 spectrophotometer (Thermo Fisher Scientific) and an Agilent Bioanalyzer 2,100 (Agilent Technologies, Santa Clara, CA, United States) to evaluate the quality of the RNA. High-quality RNA was then used to build a cDNA library following the manufacturer’s instructions. Paired-end sequencing was performed on the DNBSEQ platform (BG I, Shenzhen, China), generating 150-bp paired-end reads.

### Estimation of genome size and ploidy

SOAPnuke V1.6.5 (Chen et al., 2018b) was employed to filter out PCR duplications, low-quality reads (≥ 10% of nucleotides with a quality score ≤ 20 or the proportion of N is greater than 1%) and adapter sequences, with the following parameters: -n 0.01-l 20-q 0.1-i -Q 2-G -M 2-A 0.5 –d. Next, the software Jellyfish v2.1.4 (Marçais and Kingsford, 2011) was used to count *k*-mers of length 17–31. GenomeScope 2.0 (Ranallo-Benavidez et al., 2020) was then applied for the estimation of genome size and other features such as heterozygosity and repetition rate. Smudgeplot (Ranallo-Benavidez et al., 2020) was used for the estimation of ploidy.

### Genome assembly and evaluation

The PacBio SMRT-Analysis^2^ was used as quality control to eliminate adaptors and low-quality short reads, producing a total of 51.7 G bases (~ 72 × coverage) of PacBio HiFi data. The initial assemblies were then performed in HiFi-asm v0.15.2 (Cheng et al., 2021). To acquire high-quality, chromosome-level assemblies, Hi-C reads were compared to the contigs assembled above using Juicer (Durand et al., 2016b). Unique mapped reads with map-quality scores > 40 were subsequently used for Hi-C association chromosome assembly using the 3D-DNA pipeline (Dudchenko et al., 2017). Scaffolds were then manually checked and refined with Juicebox (Durand et al., 2016a) and visualized in Hicplotter (Akdemir and Chin, 2015). A BUSCO analysis was conducted to determine gene/genome completeness using BUSCO v4 (Simão et al., 2015) together with the embryophyta_odb10 database with 1,614 plant single-copy orthologues. BWA (Li, 2013) was used to map short reads of DNBSEQ data against the assembly. SAMtools (Danecek et al., 2021) was then employed to create a pile-up file summary of the aligned reads, and the results were imported to BCFtools (Danecek et al., 2021) for SNP and INDEL calling. The heterozygosity was then calculated as the proportion of the heterozygous sites to the total sites.

### Genome annotation

Several different methods were employed to annotate the repetitive sequences. First, Tandem Repeats Finder v4.09 (Price et al., 2005) was used for the identification of tandem repeats. Then, RepeatProteinMask v4.07 and RepeatMasker v4.07 (Chen, 2004) were used with their default parameters against RepBase v21.12 (Bao et al., 2015) to identify known repeats in a homology-based approach. Thirdly, RepeatMasker (Bedell et al., 2000) identified repeat elements with a *de novo* library, built in RepeatModeler (Abrusán et al., 2009) and LTR_FINDER v1.06 (Zhao and Hao, 2007).

To annotate the protein-coding genes, we combined RNA-based, homology-based and *de novo* methods. For the RNA-based method, we generated 63.27 million raw reads (9.49 Gb) with DNBSEQ sequencing (Supplementary Table S1). After quality control and filtering by fastp (Chen et al., 2018a), 9.48 Gb clean data were retained and aligned to scaffolds using hisat2 v2.2.1 (Kim et al., 2019). Reference genome-guided transcriptome assemblies were then constructed with StringTie v2.2.0 (Pertea et al., 2015). For homology-based predictions, the *Q. variabilis* genome was aligned against the *Arabidopsis thaliana, Q. lobata, Q. robur* and *Q. suber* genomes using TBLASTN v2.2.26 (Mount, 2007) with an *E*-value cutoff of 1*e*–5. Finally, GeneWise v2.4.1 (Birney et al., 2004) was employed for structural inspection of these alignments. For *ab initio* gene prediction, MAKER v3.01.03 (Holt and Yandell, 2011) was used to compute annotation edit distance (AED) for each protein-coding gene, based on transcript assembly from the transcriptome data, as well as from homologous annotations of the four genomes. Augustus v3.4.0 (Stanke et al., 2008; Keller et al., 2011) and SNAP (Johnson et al., 2008) were then employed for *ab initio* gene prediction using model training, based on coding sequences of 1,200 genes with structural integrity selected based on AED. Finally, the predictions obtained using these methods were combined using EVM v1.1.1 (Haas et al., 2008). The predicted genes were functionally annotated using seven public biological databases: NR, TrEMBL (Boeckmann et al., 2003), SwissProt (Boeckmann et al., 2003), KEGG (Kanehisa and Goto, 2000), InterPro (Zdobnov and Apweiler, 2001), KOG (Koonin et al., 2004), and GO (Consortium, 2004). Blast v2.2.26 was used for homolog searches with an E-value cutoff of 1e-5, and InterproScan v5.55 (Jones et al., 2014) was used for protein function prediction based on the conserved protein domains.

Homology-based non-coding RNA (ncRNA) was identified using Infernal v1.14 (Nawrocki and Eddy, 2013) by mapping plant small nuclear RNA (snRNA) and microRNA (miRNA) genes from the Rfam database (Kalvari et al., 2018). Transfer RNAs (tRNAs) were detected with tRNAscan-SE v1.3.1 (Lowe and Chan, 2016). BLASTN was used for the identification of ribosomal RNAs (rRNAs) by alignment with known plant rRNA sequences (Vitales et al., 2017).

### Genomic evolution and whole genome duplication (WGD) analysis

OrthoFinder v2.5.41 (Emms and Kelly, 2019) was used to identify homologous gene families among the assembled genomes of *Q. variabilis* and 13 further representative flowering plant species (*Amborella trichopoda*, *Arabidopsis thaliana*, *Castanea crenata*, *Castanena mollissima*, *Eucalyptus grandis*, *Juglans regia*, *Oryza sativa*, *Prunus persica*, *Q. lobata*, *Q. robur*, *Q. suber*, *Vitis vinifera* and *Xanthoceras sorbifolia*). GO enrichment analysis was conducted using ClusterProfiler with an adjusted *p* value cutoff of 0.05 (Wu et al., 2021). We performed collinearity analysis of homologous gene pairs between *Q. lobata, Q. robur*, and *Q. variabilis* using MCScanX (Wang et al., 2012).

For phylogenetic analysis and estimation of species divergence time, MUSCLE (Edgar, 2004) was applied to align the amino acid sequences of single-copy orthologous genes. The concatenated amino acid sequences were further used for construction of the phylogenetic tree in IQ-TREE2 (Minh et al., 2020). MCMCTREE of PAML (Yang, 2007) was used to estimate phylogenetic dating using a BRMC method (Sanderson, 2003) with the soft fossil calibrations obtained from the TimeTree website^3^: split of *A. trichopoda* from *O. sativa*, 173–199 million years ago (MYA); split of *X. sorbifolia* from *A. thaliana,* 96–104 MYA; split of *A. thaliana* from *J. regia,* 107–135 MYA; split of *V. vinifera* from *A. thaliana,* 89–113 MYA. Gene families were filtered out if more than 200 genes were present in one species but only 2 or fewer in the other species. The remaining gene families were used to infer the expansions and contractions of protein family in CAFÉ v3.0 (Han et al., 2013).

Searches for putative paralogous genes were conducted for *Q. variabilis* and *P. persica* against each other using BLASTP (*E*-value ≤ 1*e*–5). Syntenic blocks were then identified using MCScanX (Wang et al., 2012) with parameters of –*a* –*e* 1*e*–5–s 5. Synonymous substitutions per synonymous site (*Ks*) values were calculated with codeml in the PAML package (Yang, 2007). For interspecific orthologues, the protein sequences of the homologous genes in *Q. variabilis*, *Q. robur,* and *Q. lobata* were aligned in BLASTP (E-value ≤1e-5), and the results were sorted according to their bit-scores and E-values to obtain reciprocal optimal gene pairs. Then codeml was used to calculate the *Ks* values of reciprocal optimal gene pairs. Finally, the *Ks* distributions of intraspecific paralogs and interspecific orthologues were evaluated to infer whole genome duplication (WGD) events and divergence time in the species genome.

## Results

### Chromosome-level genome assembly

**Figure 1:**
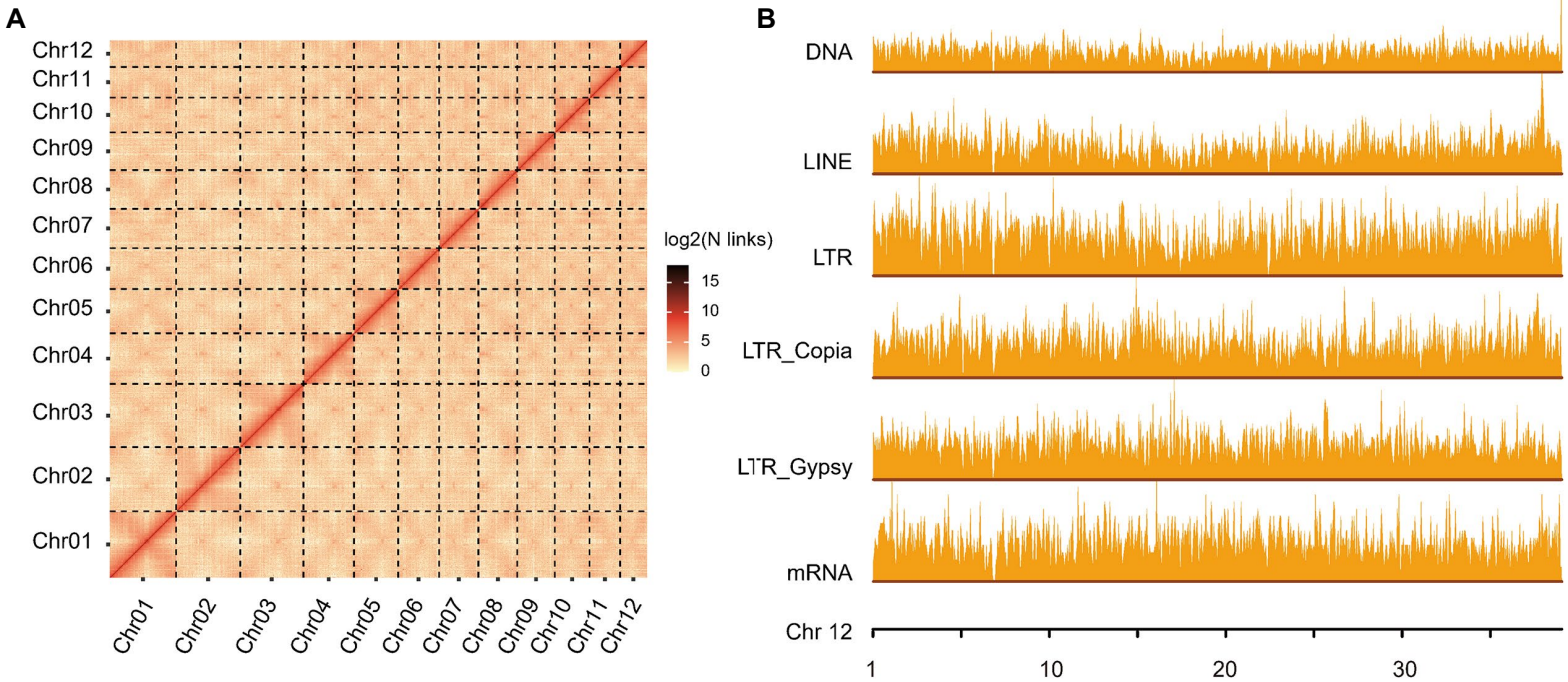
Overview of *Quercus variabilis* genome assembly and annotation. **(A)** Genome-wide chromatin interaction analysis of the *Q. variabilis* genome based on Hi-C data. **(B)** The distribution of gene and repeat sequences (DNA, LINE, LTR_Copia, LTR_Gypsy, and LTR_other) across Chr12. The height of the bars represents the distribution density of these categories and the window size was set to 50 kb.

We sequenced the *Q. variabilis* genome using a combination of PacBio and Hi-C technologies, and obtained a high-quality diploid reference genome (Smudgeplot, Supplementary Figure S1). A 20 kb DNA library was constructed and sequenced on a PacBio Sequel II platform, generating 51.70 Gb HiFi reads, approximately 72 × the estimated genome size (713.93 Mb; Supplementary Figure S2; Supplementary Table S1). Then, initial genome sequences spanning 796.30 Mb (327 contigs, N50 of 26.04 Mb; Supplementary Table S2) were constructed, slightly larger than the total genome size as estimated at 713.93 Mb using the 21-mer peak and distribution from DNBSEQ data (Supplementary Figure S2; Supplementary Table S1). This is perhaps due to chimerism caused by the relatively high heterozygosity (estimated to be 2.15%; Supplementary Figure S2). The contig N50 of *Q. variabilis* is significantly higher than that of other published congeneric species, e.g., *Q. acutissima* (1.44 Mb; Fu et al., 2022), *Q. mongolica* (2.64 Mb; Ai et al., 2020), *Q. robur* (0.07 Mb; Plomion et al., 2018), *Q. lobata* (1.9 Mb; Sork et al., 2022) and *Q. suber* (0.08 Mb; Ramos et al., 2018; Table 1). We next used 3D-DNA derived from the Hi-C data (Supplementary Table S1) to generate 12 pseudo-chromosomes (787.15 Mb, Supplementary Table S2), with lengths ranging from 39.05 to 97.21 Mb (Figure 1A; Supplementary Table S3). Interestingly, the number of pseudo-chromosomes of the assembled haploid is the same as that of other *Quercus* genomes (*Q. acutissima*, *Q. mongolica*, *Q. robur*, *Q. lobata*, and *Q. suber*). The chromosomal genome of *Q. variabilis* was characterized by 245 scaffolds, with a scaffold N50 of 64.86 Mb which is similar to that of *Q. mongolica* (66.7 Mb), slightly smaller than that of *Q. lobata* (75 Mb), but ~ 22-fold, ~ 50-fold and ~ 130-fold larger than those of *Q. acutissima*, *Q. robur*, and *Q. suber*, respectively (Table 1). We calculated the heterozygosity based on the 10,014,769 heterozygous sites (including SNPs and INDELs), and found that the heterozygosity of this genome was 1.26%, which was slightly lower than estimated (2.15%) due to underestimation (considering only SNPs and INDELs). We further evaluated the completeness of the genome assembly using the BUSCO.v4 plant datasets, and identified 1,587 (98.3%) of the 1,614 plant single-copy orthologues, with 1,526 (94.5%) presented as single-copy (Supplementary Table S4), a value superior to that of *Q. lobata* (95%)*, Q. robur* (91%), *Q. suber* (95%), *Q. acutissima* (91%) and *Q. mongolica* (92.71%), indicating that our genome assembly is of high quality and nearly complete.

**Table 1:** The statistics for genome assembly of six *Quercus* species.

	*Q. variabilis*	*Q. acutissima*	*Q. mongolica*	*Q. robur*	*Q. lobata*	*Q. suber*
Sequencing platform	DNBSEQ, Pacbio Sequel II, Hi-C	PacBio, 10X Genomics	Illumina,PacBio, Hi-C	Illumina, Roche 454	Illumina, PacBio, Hi-C	Illumina
*Assembly*						
Assembly level	Chromosome	Chromosome	Chromosome	Chromosome	Chromosome	Scaffold
Total contig length (Mb)	796	756	810	790	^*^	934
Number of contigs	327	770	645	22,615	^*^	36,760
N50 of contigs (Mb)	**26**	1.44	2.64	0.07	1.9	0.08
Total scaffold length (Mb)	796	758	810	814	847	953
Number of scaffolds	245	388	330	1,409	2,014	23,344
N50 of scaffolds (Mb)	64.9	2.9	66.7	1.3	**75**	0.5
Number of chromosomes	12	12	12	12	12	^*^
Total chromosome length (Mb)	787	750	775	717	811	^*^
% Sequence anchored on chromosome	**99**	**99**	96	96	96	0
Complete BUSCOs (%)	**98**	91	93	91	95	95

* Data not shown in the original articles; numbers in bold represent the best in each category.

Information on the genome assemblies of *Q. acutissima, Q. mongolica, Q. robur, Q. lobata*, and *Q. suber* was taken from previous reports (Plomion et al., 2018; Ramos et al., 2018; Ai et al., 2020; Fu et al., 2022; Sork et al., 2022).

### Genome annotation and gene prediction

The total length of the repetitive sequences in the *Q. variabilis* genome was 538.34 Mb, covering 67.6% of the assembled genome (Supplementary Table S5). This proportion was higher than that observed in the *Q. mongolica* genome (435.34 Mb, ~53.75% of the genome) identified using the same process (Ai et al., 2020), and also higher than those in *Q. lobata* (54%; Sork et al., 2022), *Q. suber* (51%; Ramos et al., 2018), and *E. grandis* (55%; Myburg et al., 2014), which have been calculated using other processes. TEs accounted for 61.09% of the *Q. variabilis* genome (Supplementary Table S6). Long terminal repeat retrotransposons (LTR-RT), which often contribute to variations in genome size (Feschotte et al., 2002; Du et al., 2010), were identified as being the most abundant repeats (46.63%), followed by long interspersed nuclear elements (LINE; 8.14%) and DNA elements (17.88%; Figure 1B; Supplementary Figures S3–S13; Supplementary Table S6).

**Figure 2:**
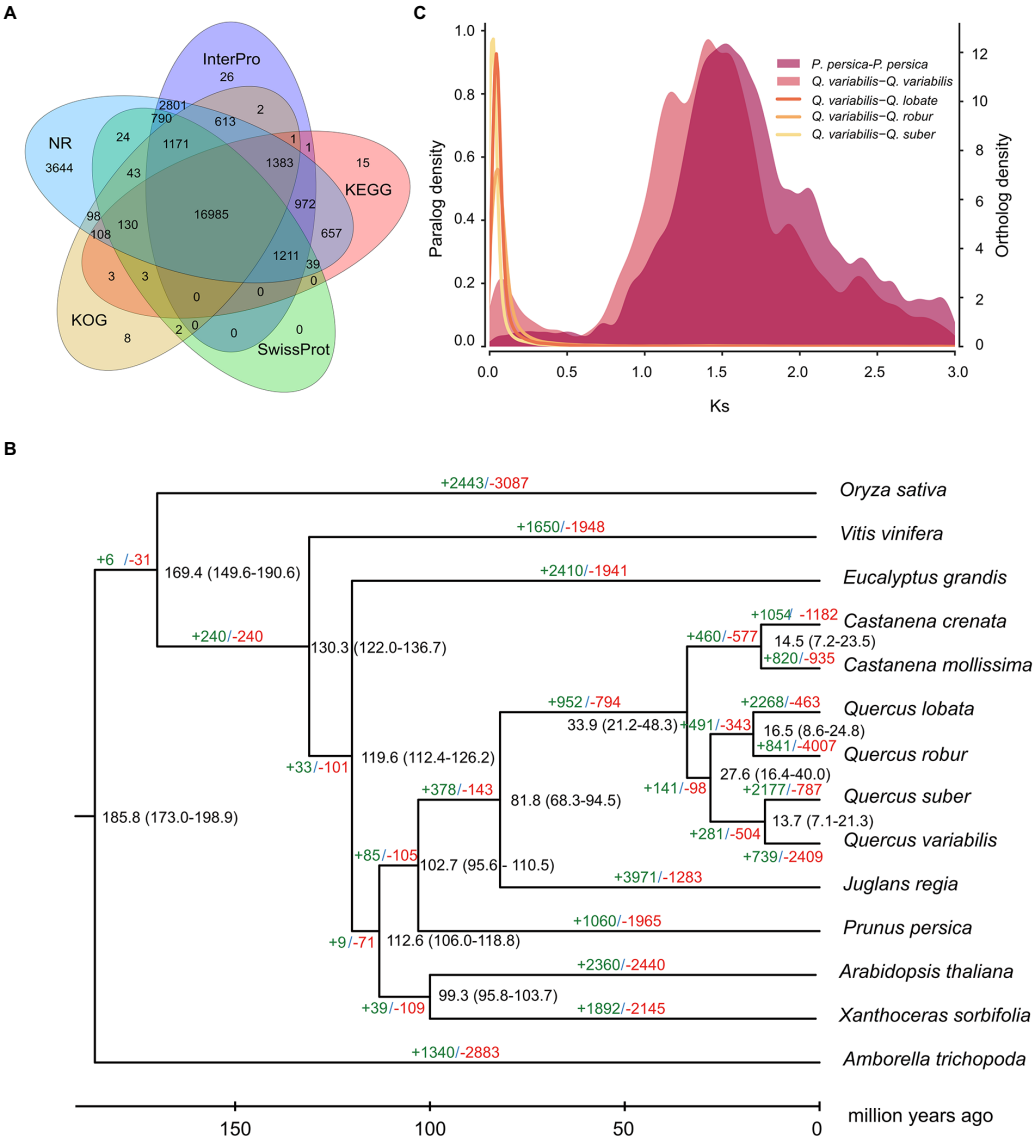
Genome functional annotation and evolution of *Q. variabilis*. **(A)** Venn diagram showing shared and unique gene functional annotations among InterPro, KEGG, SwissProt, KOG and NR databases. **(B)** Phylogenetic tree, divergence time and gene family expansion and contraction among 14 plant species. **(C)** *Ks* distribution of *Q. variabilis-Q. lobata, Q. variabilis-Q. robur, Q. variabilis-Q. suber, Q. variabilis-Q. variabilis* and *P. persica-P. persica* based on orthologous and paralogous gene pairs.

Gene models for the *Q. variabilis* genome were obtained using a comprehensive approach including *ab initio*, RNA sequence-based and homology-based predictions (Supplementary Table S7). In total, we predicted 32,466 protein-coding genes with an average gene length of 5,272.04 bp, an average coding-sequence length of 1,139.49 bp, an average exon length of 226.50 bp and an average exon number per gene of 5.03 (Supplementary Table S7). Functional annotation using the NR, Swissprot, KEGG, KOG, TrEMBL, Interpro and GO databases allowed 30,878 (95.11%) of the total 32,466 genes to be assigned putative functions (Supplementary Table S8). Of these, 52.32% (16,985) of the total genes could be functionally annotated through NR, InterPro, KEGG, SwissProt and KOG simultaneously (Figure 2A). We also predicted 12,220 rRNA, 942 tRNA, 157 miRNA, and 1,148 snRNA genes in the *Q. variabilis* genome (Supplementary Table S9).

### Orthologous gene families

**Figure 3:**
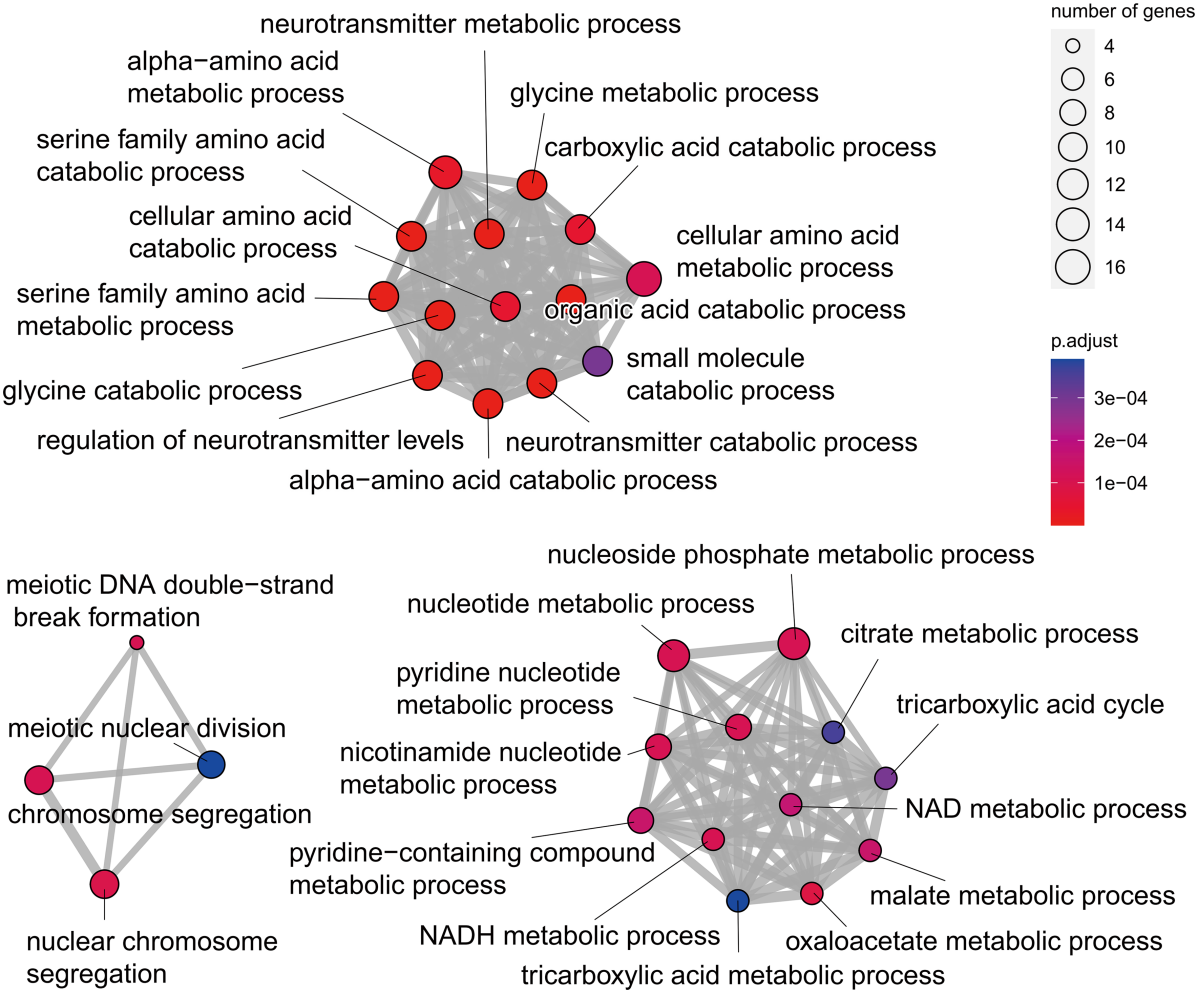
Gene ontology (GO) functional enrichment analysis of the unique genes of *Q. variabilis*.

Orthologous gene families were identified using the proteomes of *Q. variabilis* predicted in our project and those of 13 other flowering plant species, including the three congeneric species (*Q. lobata, Q. robur* and *Q. suber*; Supplementary Table S10). In total, the 29,808 *Q. variabilis* genes (91.81% of the total) clustered into 14,930 gene families, of which 6,245 gene families (including 11,113 *Q. variabilis* genes) were shared among all the 14 plant species. We also found that 964/4,854, 747/3,644, 1,821/12,372, 37/95, 891/6,186, 206/1,814, 2,486/12,784, 438/1,633, 340/891, 129/370, 4,311/11,160, 326/2,693, 814/3,015, 286/938 gene families/genes appeared to be unique to *A. trichopoda*, *A. thaliana*, *C. crenata*, *C. mollissima*, *E. grandis*, *J. regia*, *O. sativa*, *P. persica*, *Q. lobata*, *Q. robur*, *Q. suber*, *Q. variabilis*, *V. vinifera*, and *X. sorbifolia*, respectively. The gene families unique to *Q. variabilis* were mainly enriched in “glycine catabolic process,” “serine family amino acid catabolic process,” “organic acid catabolic process,” “oxaloacetate metabolic process,” “tricarboxylic acid cycle” and “nuclear chromosome segregation” (Figure 3; Supplementary File S1).

### Genome evolution

Phylogenetic analysis was conducted based on the 483 single-copy gene families derived from *Q. variabilis* and 13 further flowering plant species (Supplementary Table S10). We found that within this subclade, *Q. variabilis* is more closely related to *Q. suber* than to *Q. lobata* or *Q. robur* (Figure 2B). The divergence between *Q. variabilis* and *Q. suber* occurred at approximately 13.7 (7.1–21.3) MYA, while *Q. lobata* and *Q. robur,* which belong to a different subclade, diverged from the *Q. variabilis-Q. suber* subclade ~27.6 (16.4–40.0) MYA (Figure 2B).

**Figure 4:**
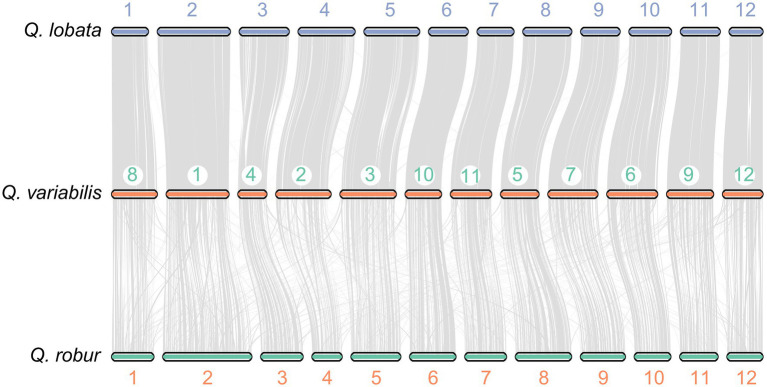
Synteny blocks identified between *Q. variabilis* and *Q. lobata*, and *Q. variabilis* and *Q. robur*.

To investigate potential WGD events in the evolutionary history of *Q. variabilis*, we studied the distribution of the *Ks* between homologous gene pairs derived from *Q. variabilis*, *Q. lobata*, *Q. robur*, *Q. suber*, and *P. persica*. One peak was found based on the paralogous gene pairs in *Q. variabilis* and *P. persica* (~ 1.5 *Ks* units), indicating a shared ancient WGD event (*γ*) for these two species (Murat et al., 2015; Figure 2C). The divergence between *Q. variabilis* and three congeneric species (*Q. lobata, Q. robur* and *Q. suber*; 0.02–0.05 *Ks* units) occurred later than the WGD event (Figure 2C). Further investigation of the genomic collinearity between *Q. variabilis* and *Q. lobata, Q. robur* showed a clear one-to-one syntenic relationship, and the overall gene synteny was largely conserved (Figure 4), suggesting that no large amounts of chromosome fusion or species-specific WGD events occurred after species divergence (Ai et al., 2020).

### Gene family expansion and contraction

We analyzed gene family expansion and contraction based on the gene families in the 14 studied flowering plant genomes (Supplementary Table S10) using OrthoFinder. The number of expanded/contracted gene families in *Q. variabilis* compared with its common ancestor were 739/2,409, while in *Q. suber*, which is genomically the most similar to *Q. variabilis,* these numbers were 2,177/787 (Figure 2B). A significant number of expanded genes in *Q. variabilis* were enriched in “monoterpene metabolic process,” “terpene biosynthetic process,” “intrachromosomal DNA recombination,” “hydrocarbon biosynthetic process” and “oxaloacetate metabolic process” (Supplementary Figure S14; Supplementary File S2), while contracted gene families were enriched in “glutathione metabolic process,” “isoflavonoid biosynthetic process,” “toxin catabolic process,” “programmed cell death induced by symbiont” and “regulation of response to red or far red light” (Supplementary Figure S15; Supplementary File S3).

## Discussion

*Quercus variabilis*, the Chinese cork oak, is an ecologically and economically valuable deciduous broadleaved tree species native to and widespread in East Asia (Fujiwara and Harada, 2015). Here, we present a chromosome-scale high-quality *de novo* genome assembly for *Q. variabilis* using a combination of PacBio Sequel II and Hi-C sequencing data. This *Q. variabilis* genome is 796.30 Mb, of which approximately 98.85% (787.15 Mb, Supplementary Table S2) can be anchored to 12 chromosomes. The quality of the *Q. variabilis* genome assembly was higher than that of several other published *Quercus* genomes, including those of *Q. acutissima* (Fu et al., 2022), *Q. mongolica* (Ai et al., 2020), *Q. robur* (Plomion et al., 2018), *Q. lobata* (Sork et al., 2022) and *Q. suber* (Ramos et al., 2018), although the *Q. lobata* genome had a slightly longer scaffold N50 than did that of *Q. variabilis.* It is worth noting that 98.3% of the plant single-copy orthologs was detected in the assembly genome, which is a higher percentage than detected in *Q. lobata* (95%)*, Q. robur* (91%), *Q. suber* (95%), *Q. acutissima* (91%) or *Q. mongolica* (92.71%; Table 1). Altogether, the assembly of *Q. variabilis* is relatively accurate and complete. This is the first reference genome for *Q. variabilis* and will lay the foundation for understanding the evolution of this species and will provide important resources for the further investigation of genetic diversity in *Q. variabilis* and related species.

## Data availability statement

The raw sequencing data presented in the study are deposited in the NCBI SRA, BioProject No. PRJNA849150. The whole-genome sequencing data reported in the study are deposited into CNGB Sequence Archive (CNSA) (Guo et al., 2020) of China National GeneBank DataBase (CNGBdb) (Chen et al., 2020a), accession number CNP0003390, and is publicly accessible at https://db.cngb.org/.

## Author contributions

BH and D-ZL conceived and designed the study. K-HJ, YX, X-MX, W-QL, YZ, and R-GZ analyzed the data. LW wrote the manuscript. BH, D-ZL, K-HJ, and XQ edited and improved the manuscript. All authors contributed to the article and approved the submitted version.

## Funding

This study was supported by the Project funded by the Postdoctoral Science Foundation “Research and development of key technologies and equipment of germplasm bank” (BSHCX202101), the Postdoctoral Station Recruitment Subsidy of Shandong Province “Collection, preservation, evaluation and utilization of *Quercus acutissima* and *Q. variabilis* Germplasm Resources” (BSHCX202102), and the Agricultural Science and Technology Innovation Project of SAAS (CXGC2022E13).

## Conflict of interest

R-GZ was employed by Ori (Shandong) Gene Science and Technology Co., Ltd.

The remaining authors declare that the research was conducted in the absence of any commercial or financial relationships that could be construed as a potential conflict of interest.

## Publisher’s note

All claims expressed in this article are solely those of the authors and do not necessarily represent those of their affiliated organizations, or those of the publisher, the editors and the reviewers. Any product that may be evaluated in this article, or claim that may be made by its manufacturer, is not guaranteed or endorsed by the publisher.

## Footnotes

^1^ http://www.pacb.com/

^2^ https://www.pacb.com

^3^ http://www.timetree.org/
