# Supplementary material for: A chromosome-level genome assembly of the Chinese cork oak (Quercus variabilis)
Source: Front Plant Sci. 2022 Sep 23;13:1001583. doi: 10.3389/fpls.2022.1001583 (PMC9538376; doi:10.3389/fpls.2022.1001583)
Supplement: Supplementary file 1 [file Data_Sheet_1.docx]

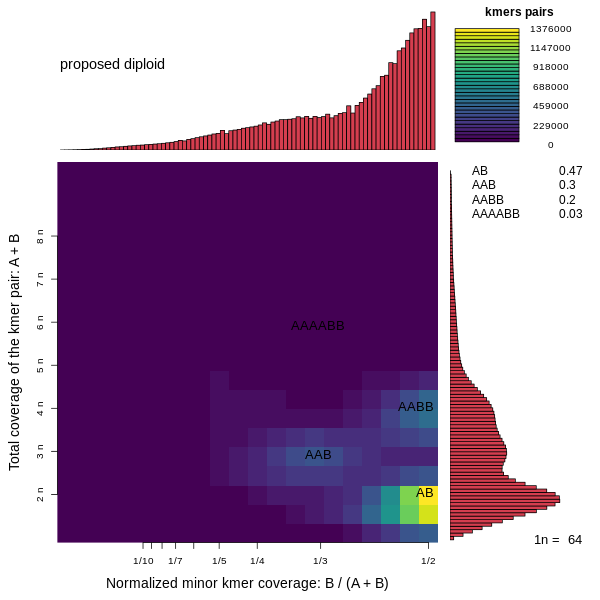


Fig. S1 Smudgeplots showing the coverage and distribution of *k*-mer pairs that fit the diploid genome model.


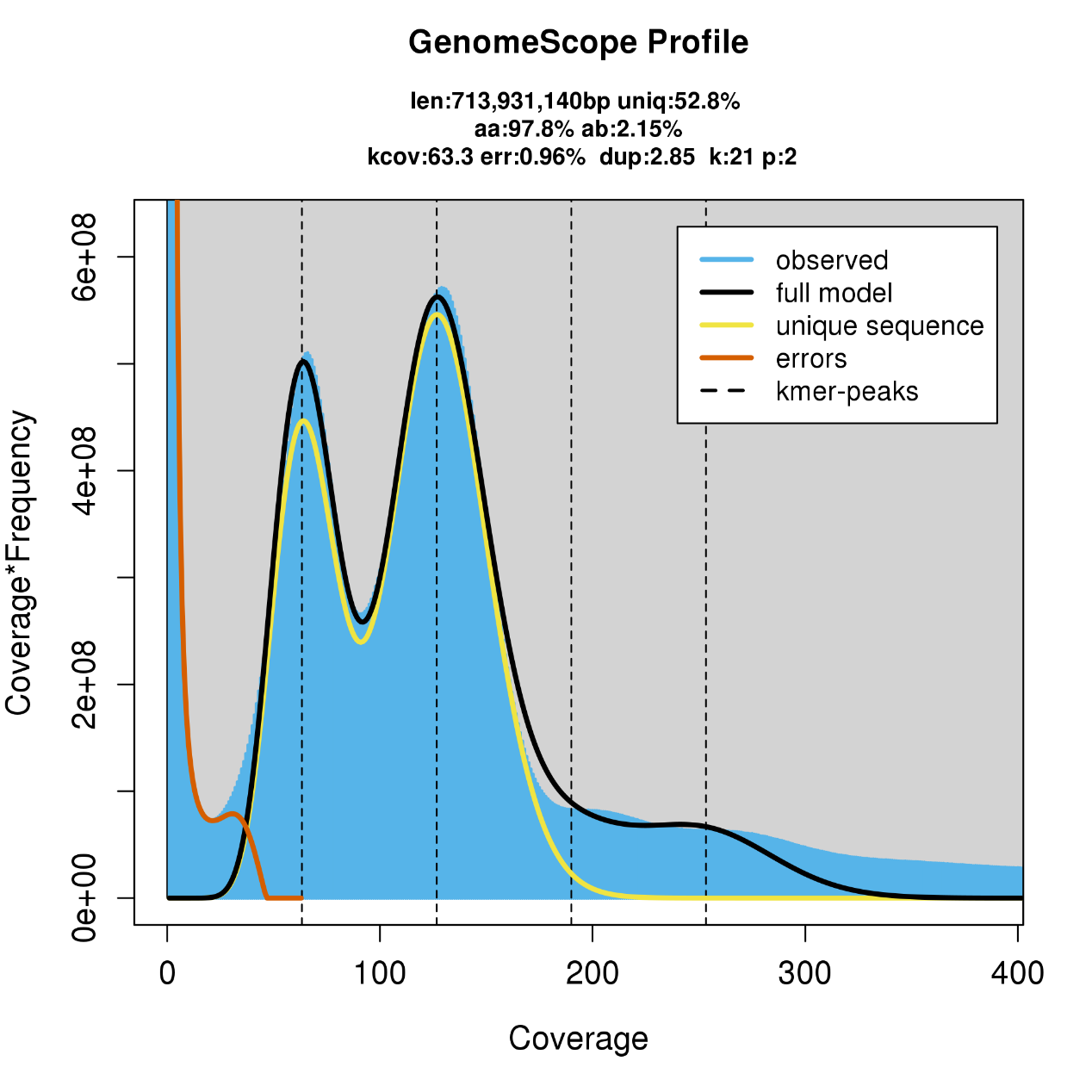


Fig. S2 Genome size estimation by 21-mer distribution.


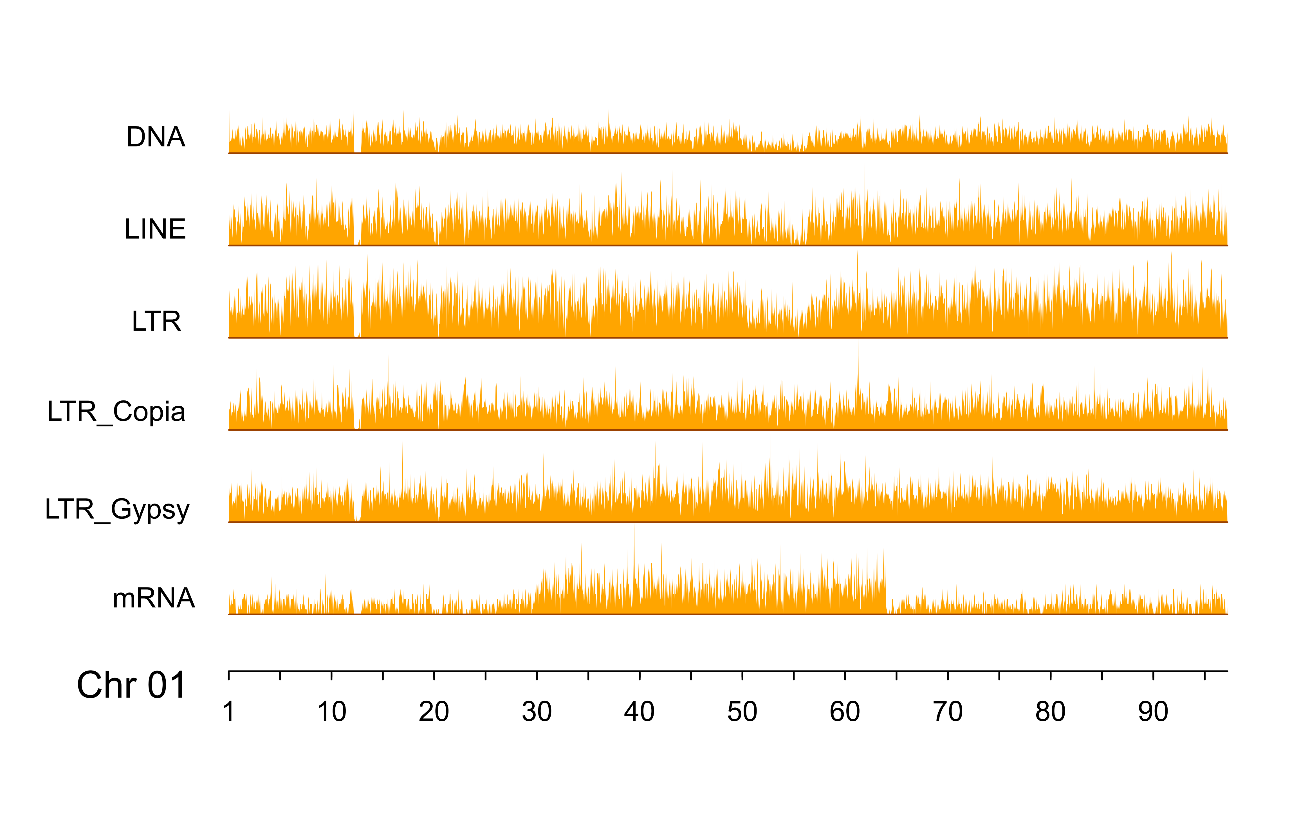


Fig. S3 The distribution of gene and several common repeat categories including DNA, LINE, LTR_Copia, LTR_Gypsy and LTR_other across Chr 01 were demonstrated using karyoploteR.


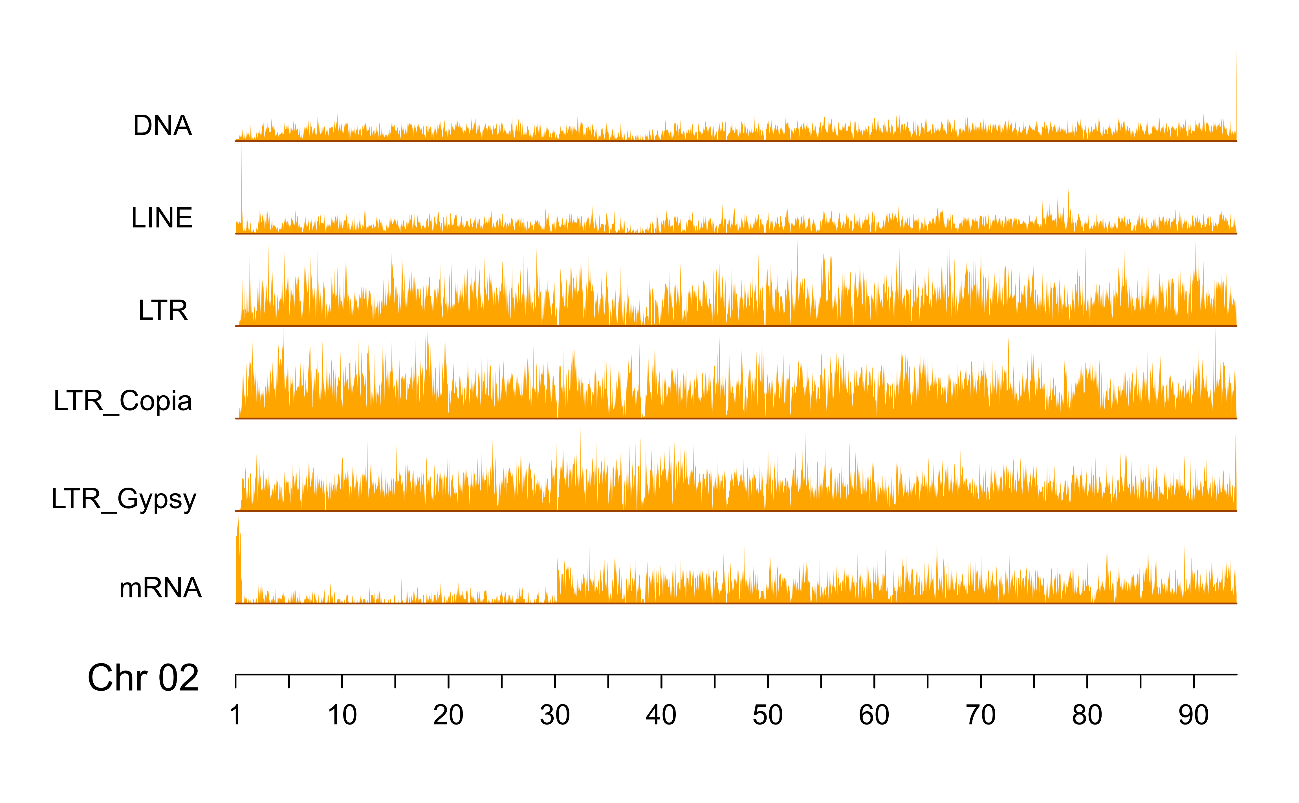


Fig. S4 The distribution of gene and several common repeat categories including DNA, LINE, LTR_Copia, LTR_Gypsy and LTR_other across Chr 02 were demonstrated using karyoploteR.


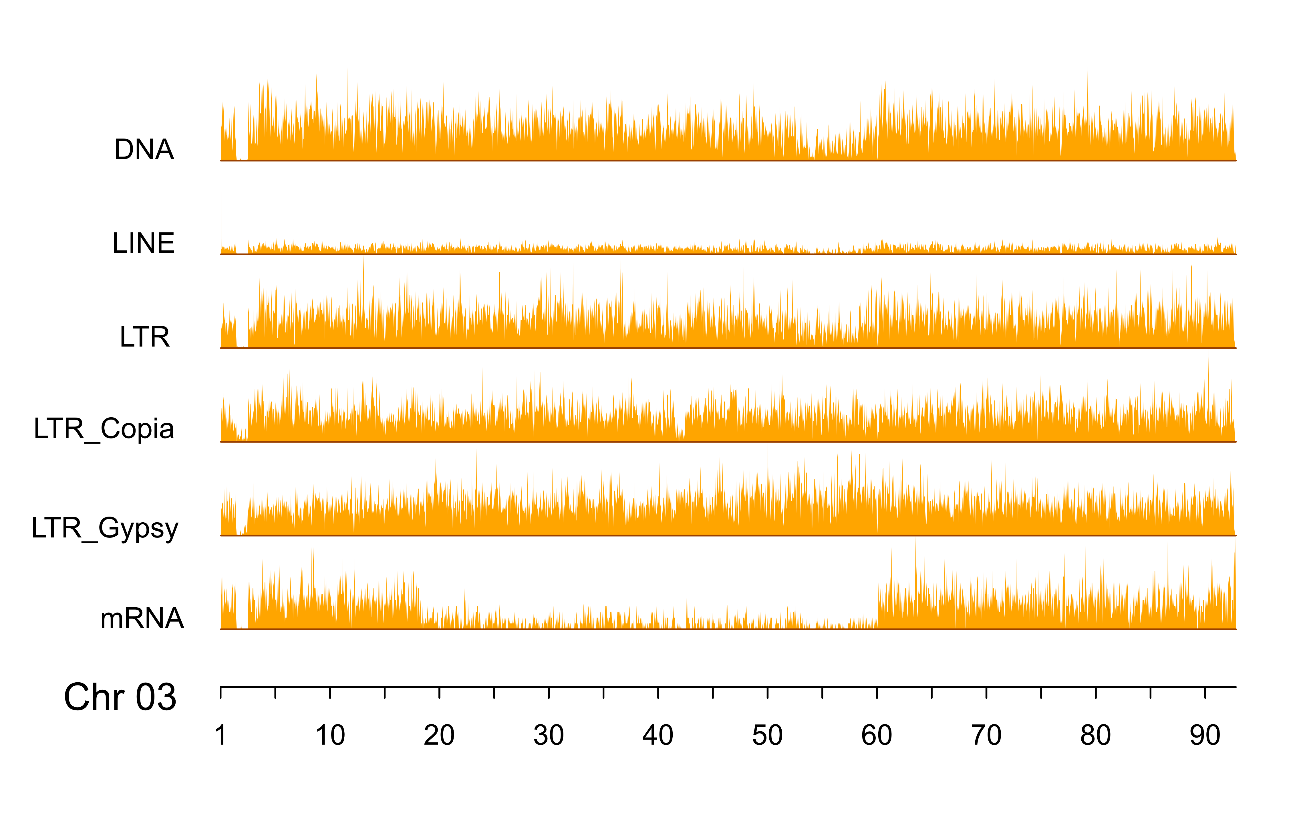


Fig. S5 The distribution of gene and several common repeat categories including DNA, LINE, LTR_Copia, LTR_Gypsy and LTR_other across Chr 03 were demonstrated using karyoploteR.


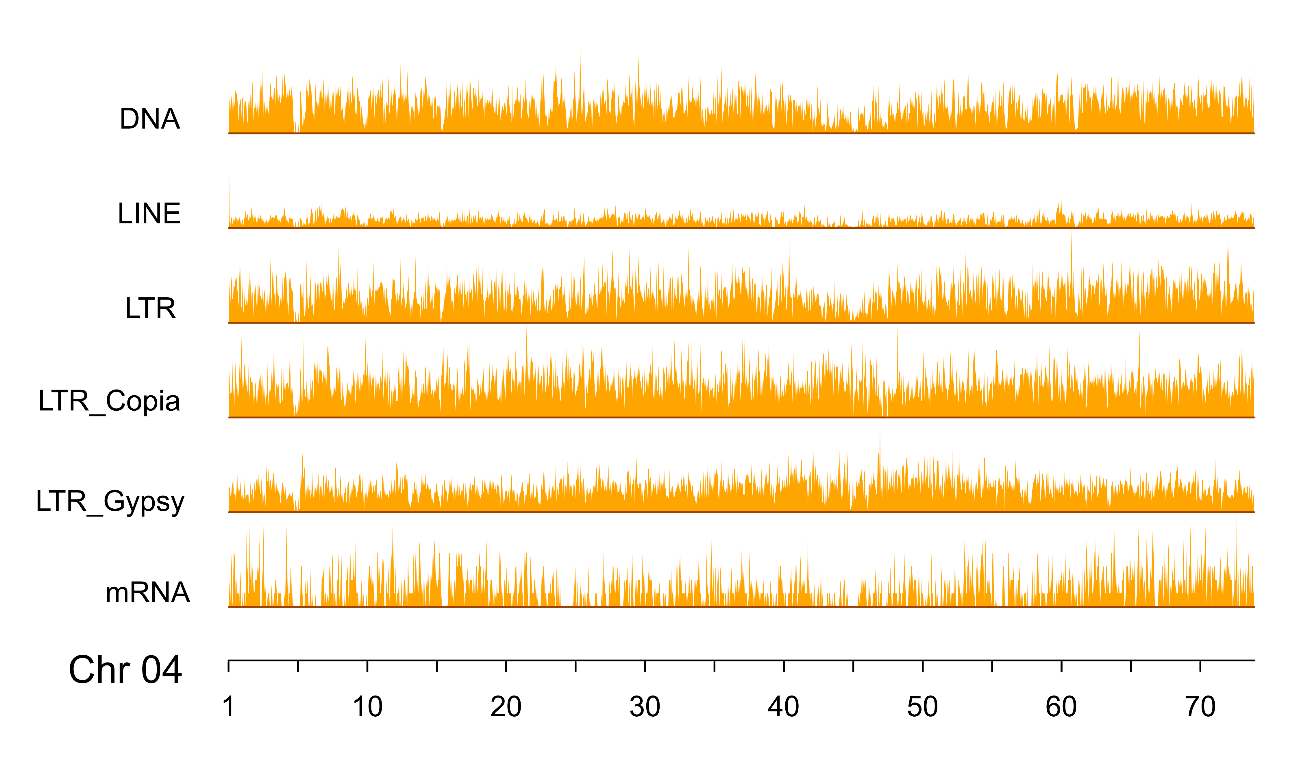


Fig. S6 The distribution of gene and several common repeat categories including DNA, LINE, LTR_Copia, LTR_Gypsy and LTR_other across Chr 04 were demonstrated using karyoploteR.


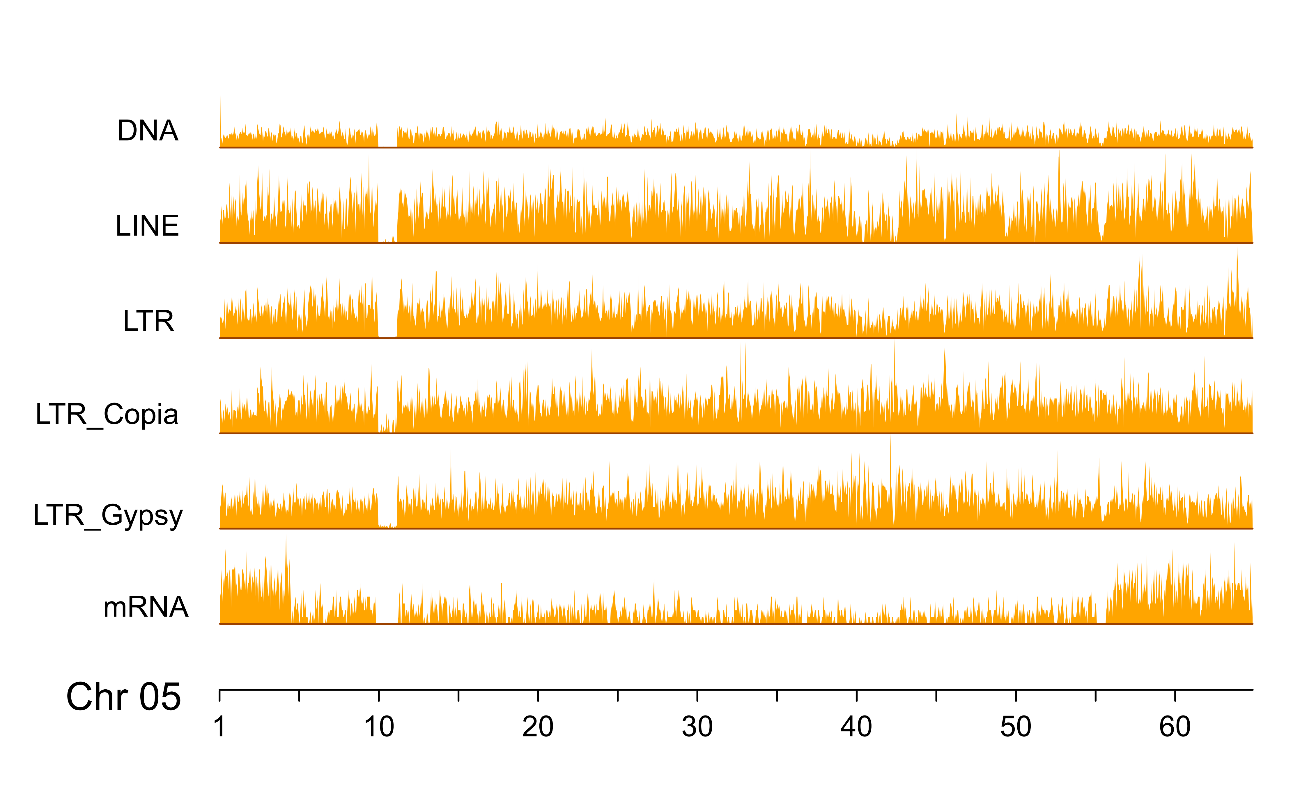


Fig. S7 The distribution of gene and several common repeat categories including DNA, LINE, LTR_Copia, LTR_Gypsy and LTR_other across Chr 05 were demonstrated using karyoploteR.


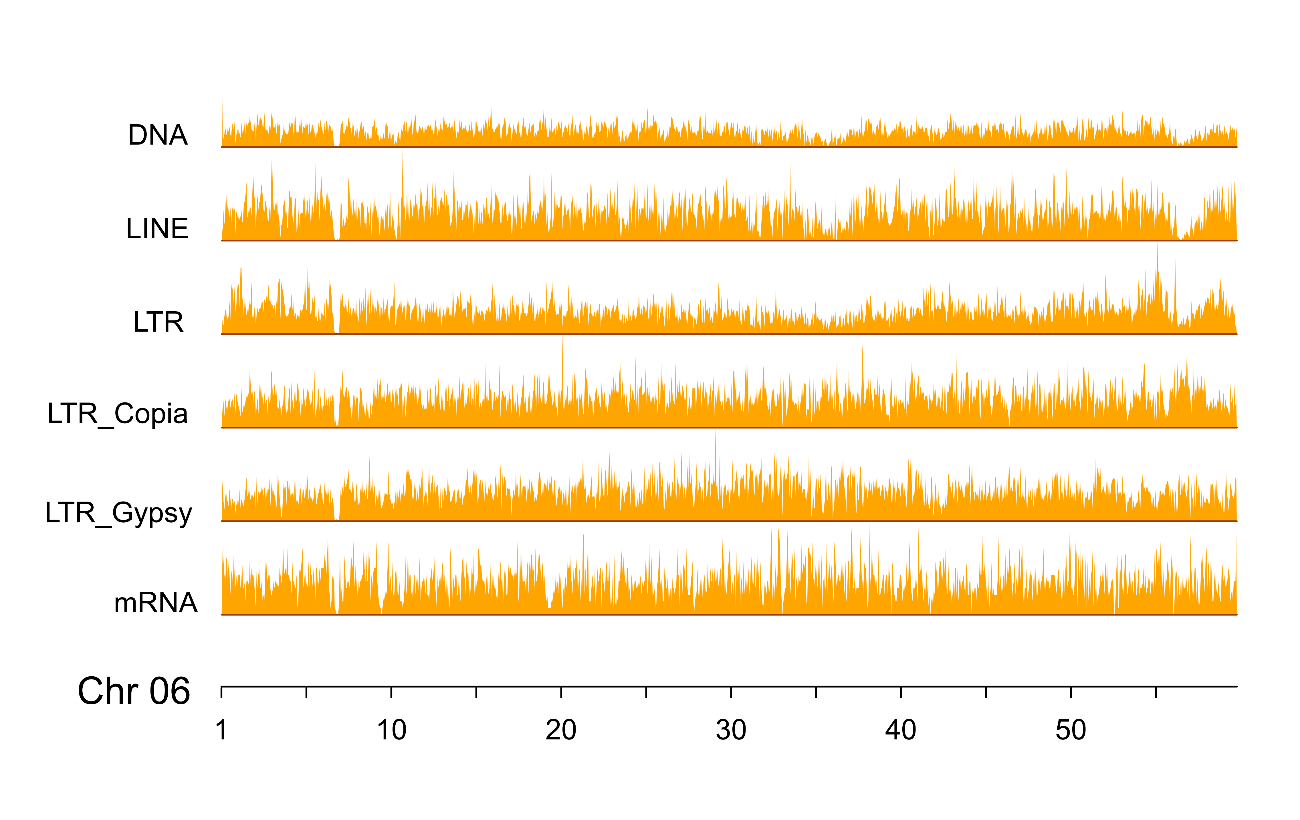


Fig. S8 The distribution of gene and several common repeat categories including DNA, LINE, LTR_Copia, LTR_Gypsy and LTR_other across Chr 06 were demonstrated using karyoploteR.


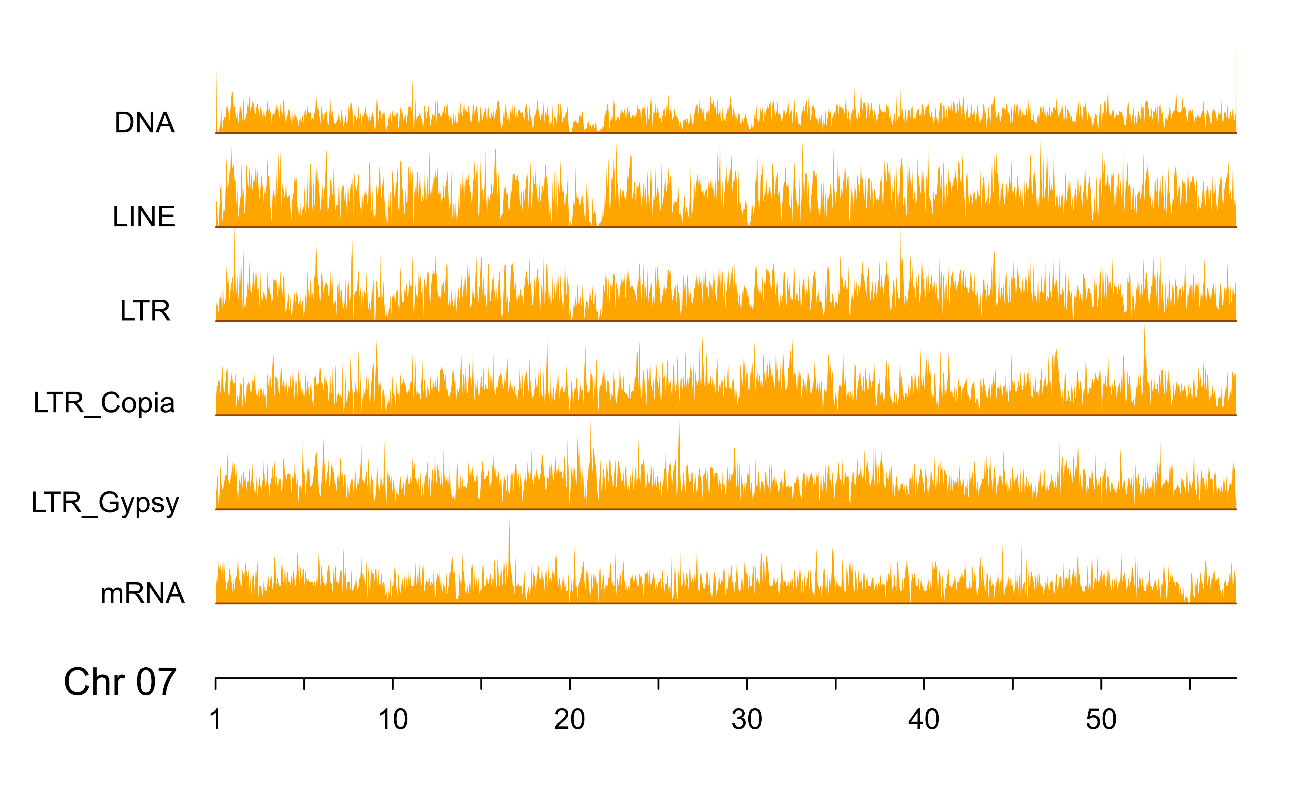


Fig. S9 The distribution of gene and several common repeat categories including DNA, LINE, LTR_Copia, LTR_Gypsy and LTR_other across Chr 07 were demonstrated using karyoploteR.


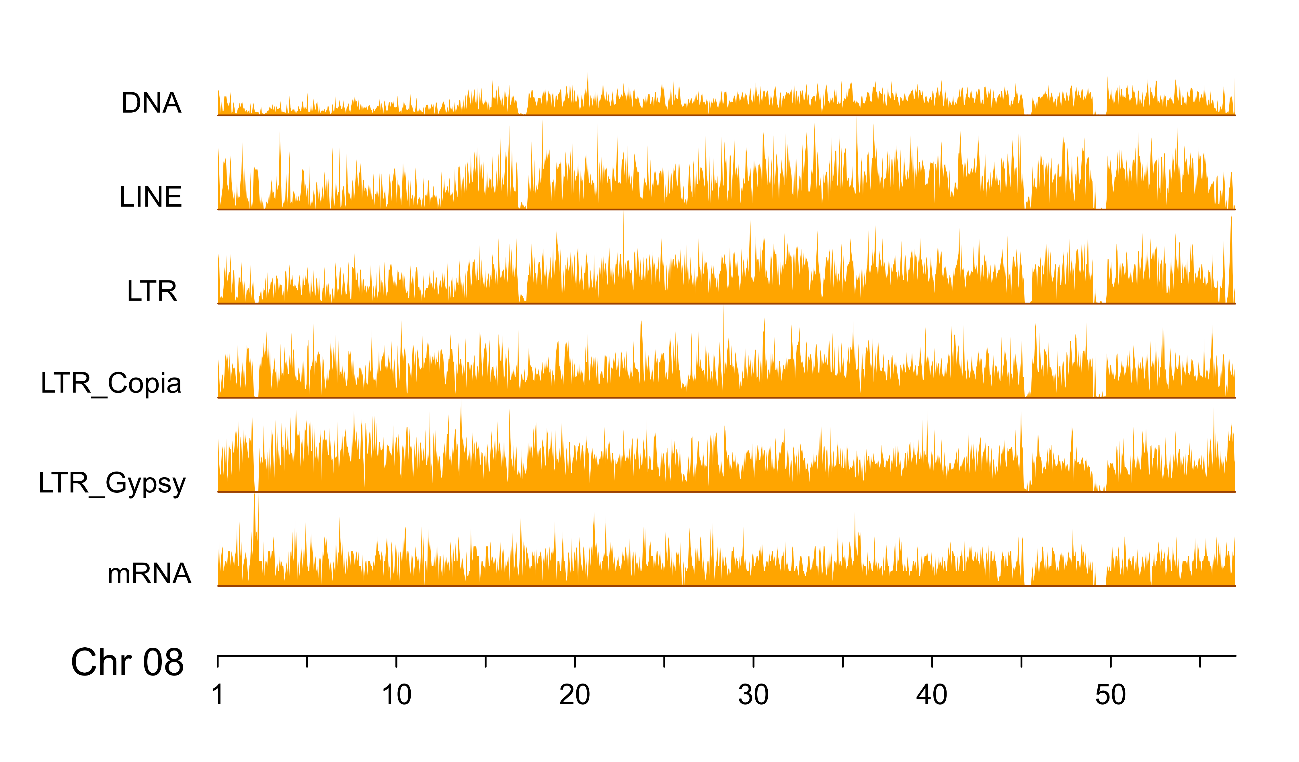


Fig. S10 The distribution of gene and several common repeat categories including DNA, LINE, LTR_Copia, LTR_Gypsy and LTR_other across Chr 08 were demonstrated using karyoploteR.


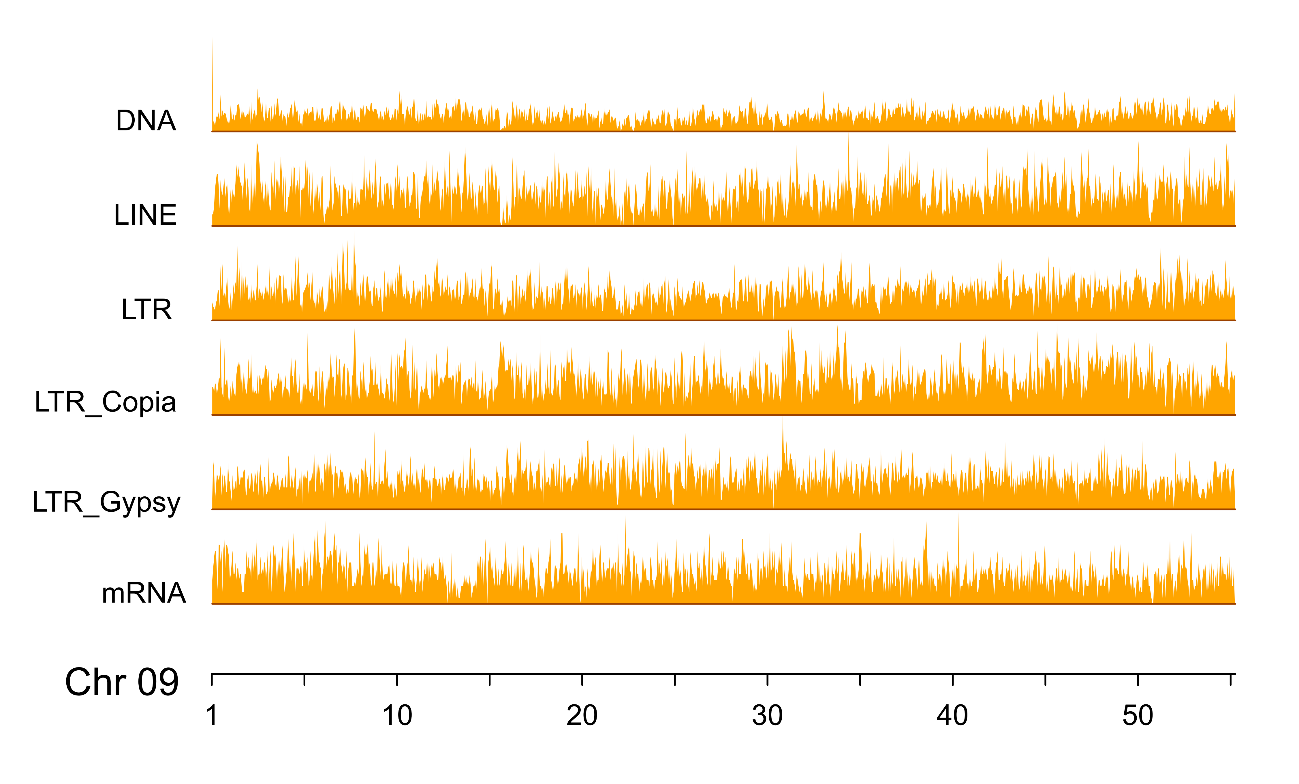


Fig. S11 The distribution of gene and several common repeat categories including DNA, LINE, LTR_Copia, LTR_Gypsy and LTR_other across Chr 09 were demonstrated using karyoploteR.


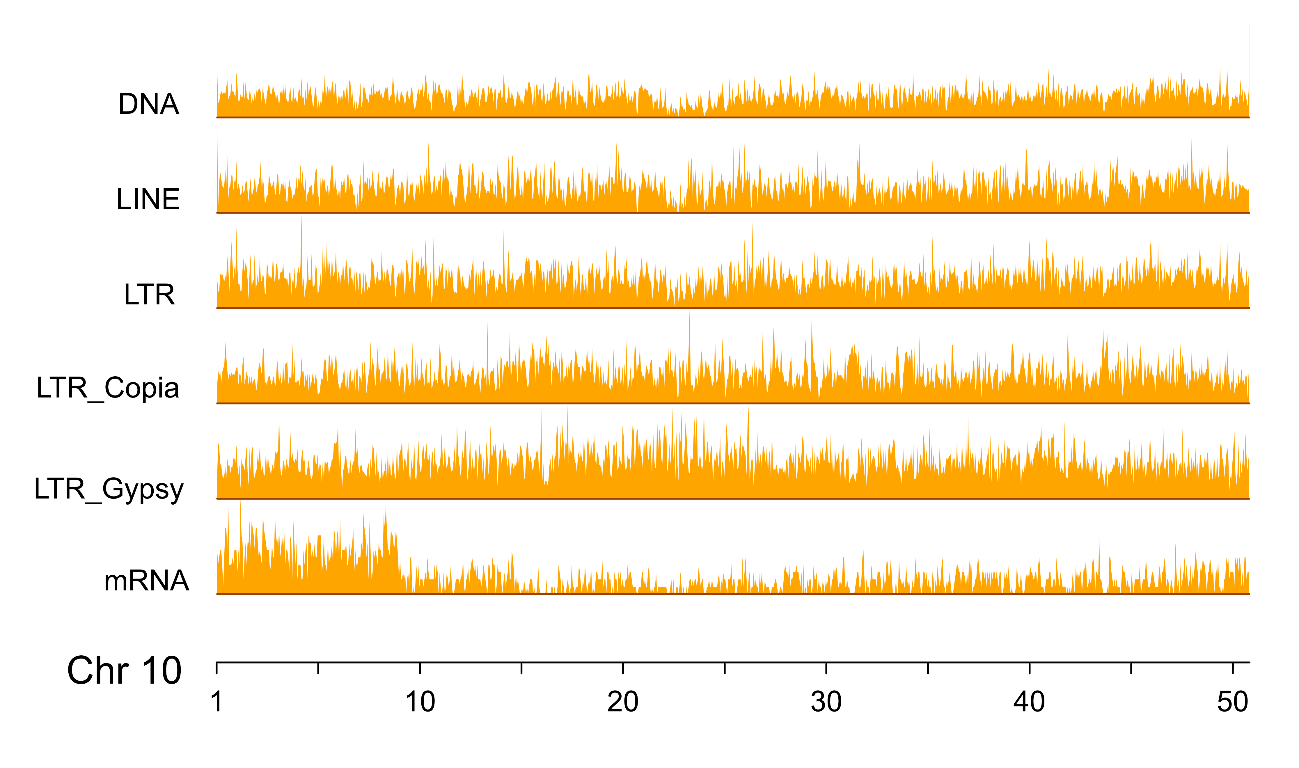


Fig. S12 The distribution of gene and several common repeat categories including DNA, LINE, LTR_Copia, LTR_Gypsy and LTR_other across Chr 10 were demonstrated using karyoploteR.


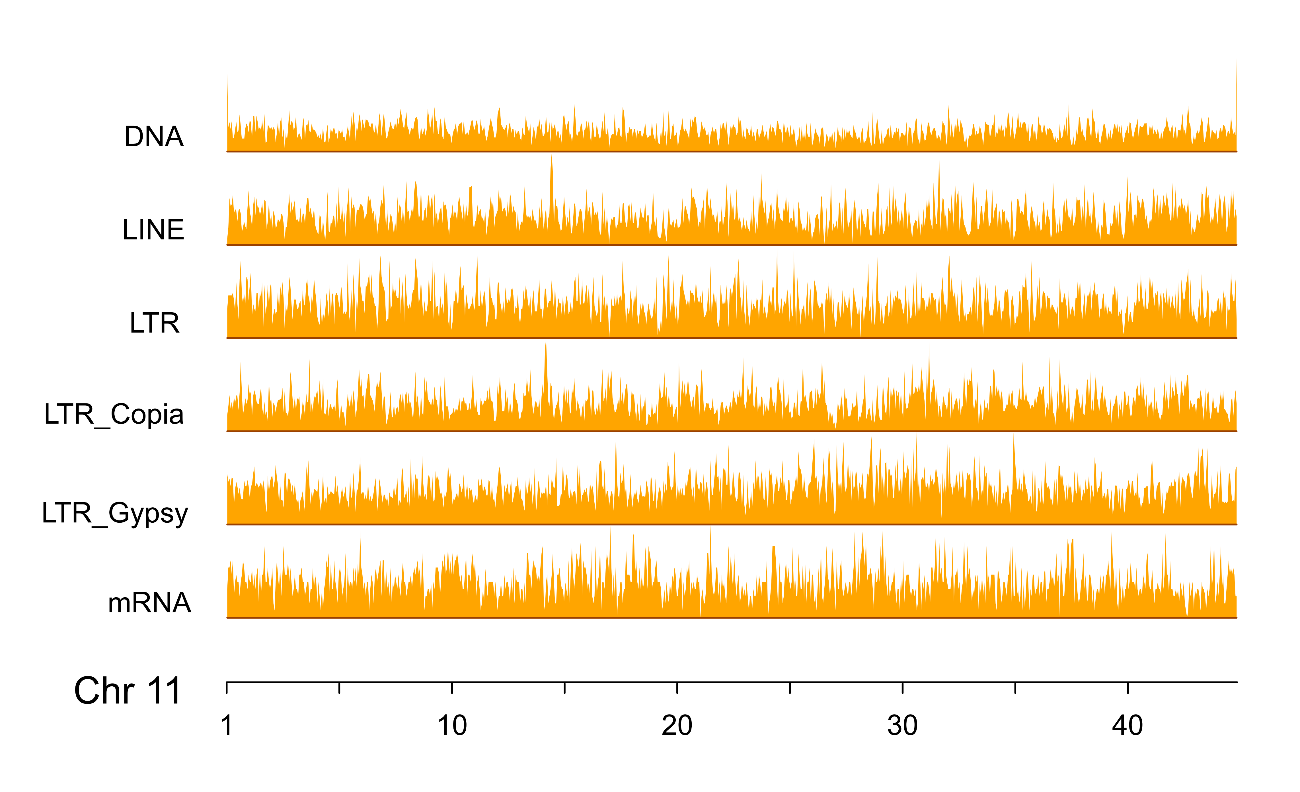


Fig. S13 The distribution of gene and several common repeat categories including DNA, LINE, LTR_Copia, LTR_Gypsy and LTR_other across Chr 11 were demonstrated using karyoploteR.


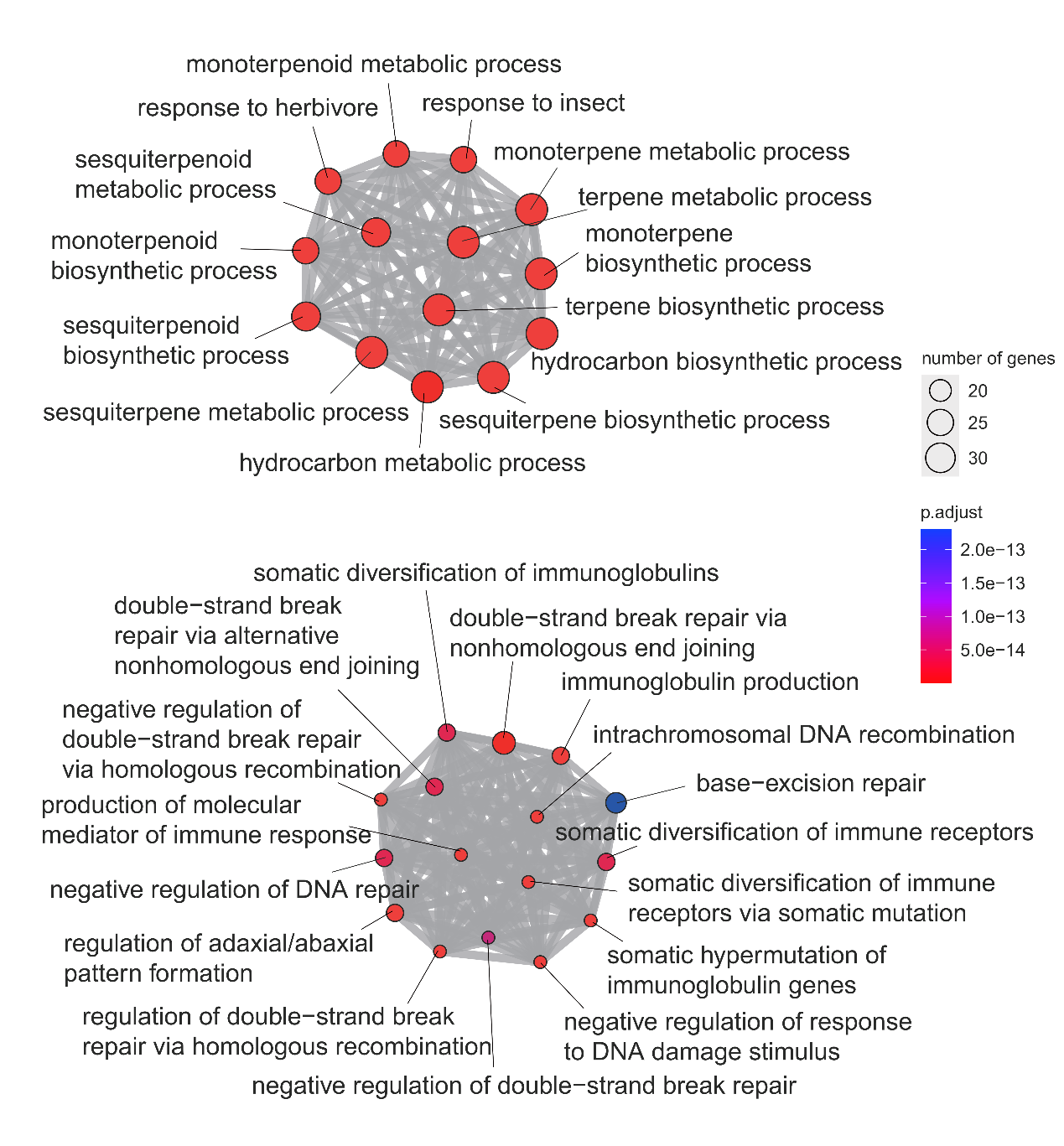


Fig. S14 GO functional enrichment analysis of the expanded gene families of *Q. variabilis*.


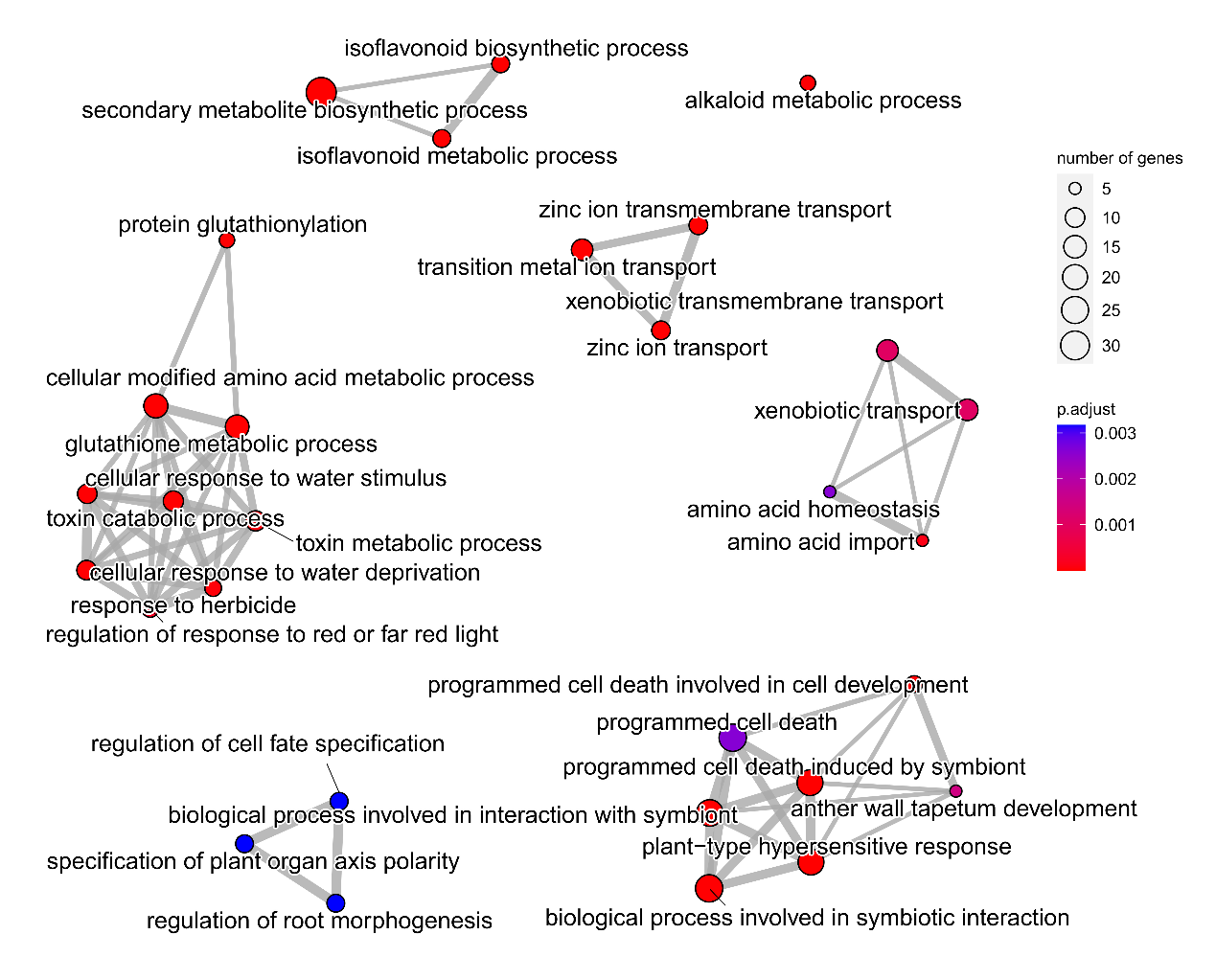


Fig. S15 GO functional enrichment analysis of the contracted gene families of *Q. variabilis*.

Table S1. Statistics of sequencing data.

| Platform | Clean reads (M) | Clean bases (Gb) |
| --- | --- | --- |
| DNBSEQ | 849.41 | 127.75 |
| Pacbio Sequel II | 4.17 | 51.7 |
| Hi-C | 859.02 | 128.85 |
| RNA-seq | 63.26 | 9.48 |

| Table S2. Summary of genome assembly of *Q. variabilis*. | | |
| --- | --- | --- |
|  | Initial assembly using HiFi data | Final assembly via Hi-C data |
| Size of assembled contigs/scaffolds (bp) | 796,297,153 | 796,338,153 |
| Number of contigs/scaffolds | 327 | 245 |
| Contig/scaffold N50 size (bp) | 26,037,807 | 64,863,055 |
| Contig/scaffold N90 size (bp) | 7,829,573 | 44,846,970 |
| Size of assembled chromosomes | / | 787,152,643 |

Table S3. Top 12 longest scaffolds.

| No. | Length (bp) |
| --- | --- |
| 1 | 97,214,915 |
| 2 | 94,031,974 |
| 3 | 92,800,393 |
| 4 | 73,893,829 |
| 5 | 64,863,055 |
| 6 | 59,757,167 |
| 7 | 57,626,006 |
| 8 | 56,994,178 |
| 9 | 55,253,568 |
| 10 | 50,818,764 |
| 11 | 44,846,970 |
| 12 | 39,051,824 |
| Total | 787,152,643 |

Table S4. Completeness evaluation of the genome assembly using BUSCO

| Type | Number | Percentage (%) |
| --- | --- | --- |
| Complete BUSCOs (C) | 1,587 | 98.3 |
| Complete and single-copy BUSCOs (S) | 1,526 | 94.5 |
| Complete and duplicated BUSCOs (D) | 61 | 3.8 |
| Fragmented BUSCOs (F) | 5 | 0.3 |
| Missing BUSCOs (M) | 22 | 1.4 |
| Total | 1,614 | 100 |

Table S5. Prediction of the repeat sequences in the *Q. variabilis* genome

| Type | Repeat Length (bp) | % of genome |
| --- | --- | --- |
| Tandem Repeat Finder | 48,904,650.00 | 6.14 |
| Repeatmasker | 84,925,641.00 | 10.66 |
| Proteinmask | 66,737,772.00 | 8.38 |
| De novo | 526,430,457.00 | 66.11 |
| Total | 538,342,432.00 | 67.6 |

Table S6. Categories of transposable elements (TEs) predicted in the *Q. variabilis* genome

| Type | | Length(bp) | % of genome |
| --- | --- | --- | --- |
| Retro | Retro/LTR/Copia | 140,049,180 | 17.5867 |
|  | Retro/LTR/Gypsy | 149,593,986 | 18.7852 |
|  | Retro/LTR/Other | 81,651,374 | 10.2534 |
|  | Retro/SINE | 295,046 | 0.0371 |
|  | Retro/LINE | 64,804,322 | 8.1378 |
|  | Retro/Other | 41,420 | 0.0052 |
| DNA | DNA/EnSpm | 31,851,651 | 3.9998 |
|  | DNA/Harbinger | 10,055,359 | 1.2627 |
|  | DNA/hAT | 34,607,135 | 4.3458 |
|  | DNA/Helitron | 12,855,314 | 1.6143 |
|  | DNA/Mariner | 14,343 | 0.0018 |
|  | DNA/MuDR | 12,502,439 | 1.5700 |
|  | DNA/P | 939,514 | 0.1180 |
|  | DNA/Other | 39,595,483 | 4.9722 |
| Other | | 3,081,179 | 0.3869 |
| Total | | 486,463,975 | 61.0876 |

Table S7. Gene annotation summary for the *Q. variabilis* genome.

| Gene set | | Number | Average gene length (bp) | Average CDS length (bp) | Average exon per gene | Average exon length (bp) | Average intron length (bp) |
| --- | --- | --- | --- | --- | --- | --- | --- |
| De novo | Snap | 265,508 | 1,638.26 | 498.93 | 2.88 | 173.45 | 607.15 |
|  | Augustus | 83,815 | 3,783.50 | 1,100.10 | 4.1 | 268.41 | 866 |
| homolog | *A. thaliana* | 123,717 | 2,595.09 | 898.19 | 3.23 | 278.07 | 760.92 |
|  | *Q. lobata* | 4,883,294 | 1,240.97 | 876.4 | 1.61 | 543.42 | 594.99 |
|  | *Q. robur* | 331,972 | 2,043.10 | 928.51 | 2.34 | 396.53 | 830.8 |
|  | *Q. suber* | 4,328,143 | 1,160.66 | 823.87 | 1.58 | 522.31 | 583.33 |
| RNA-seq | Transcript | 41,875 | 9,669.15 | 1,792.07 | 6.13 | 292.38 | 1,535.73 |
| Final | | 32,466 | 5,272.04 | 1139.49 | 5.03 | 226.50 | 1025.24 |

Table S8. Summary of gene function annotation for the *Q. variabilis* genome.

|  | Total | Nr | Swissprot | KEGG | KOG | TrEMBL | Interpro | GO | Overall |
| --- | --- | --- | --- | --- | --- | --- | --- | --- | --- |
| Number | 32,466 | 30,669 | 20,398 | 21,508 | 20,550 | 30,131 | 25,956 | 16,222 | 30,878 |
| Percentage | 100% | 94.46% | 62.83% | 66.25% | 63.3% | 92.81% | 79.95% | 49.97% | 95.11% |

Table S9. Annotation of non-coding RNAs for the *Q. variabilis* genome.

| Type | | Copy | Average length (bp) | Total length (bp) | % of genome |
| --- | --- | --- | --- | --- | --- |
| miRNA | | 157 | 117.4331 | 18437 | 0.002315 |
| tRNA | | 942 | 75.28769 | 70921 | 0.008906 |
| rRNA | rRNA | 6110 | 392.3789 | 2397435 | 0.301057 |
|  | 18S | 989 | 1682.948 | 1664436 | 0.209011 |
|  | 28S | 3611 | 142.8748 | 515921 | 0.064787 |
|  | 5.8S | 913 | 161.9869 | 147894 | 0.018572 |
|  | 5S | 597 | 115.8861 | 69184 | 0.008688 |
| snRNA | snRNA | 574 | 118.6429 | 68101 | 0.008552 |
|  | CD-box | 364 | 105.0989 | 38256 | 0.004804 |
|  | HACA-box | 45 | 123.2889 | 5548 | 0.000697 |
|  | splicing | 165 | 147.2545 | 24297 | 0.003051 |
| Total | | 14467 | / | 5020430 | 0.63044 |

Table S10 Summary of gene family clustering of the 14 plant genomes

| species | Number of genes | Number of genes in orthogroups | Number of unassigned genes | Number of species-specific orthogroups | Number of genes in species-specific orthogroups |
| --- | --- | --- | --- | --- | --- |
| *Arabidopsis thaliana* | 27569 | 25435 | 2134 | 747 | 3644 |
| *Amborella trichopoda* | 26846 | 22510 | 4336 | 964 | 4854 |
| *Castanena crenata* | 69980 | 65336 | 4644 | 1821 | 12372 |
| *Castanena mollissima* | 33597 | 33040 | 557 | 37 | 95 |
| *Eucalyptus grandis* | 35600 | 34195 | 1405 | 891 | 6186 |
| *Juglans regia* | 30567 | 30098 | 469 | 206 | 1814 |
| *Oryza sativa* | 42173 | 34233 | 7940 | 2486 | 12784 |
| *Prunus persica* | 26873 | 24543 | 2330 | 438 | 1633 |
| *Quercus lobata* | 39373 | 37856 | 1517 | 340 | 891 |
| *Quercus robur* | 25808 | 24774 | 1034 | 129 | 370 |
| *Quercus suber* | 48071 | 46100 | 1971 | 4311 | 11160 |
| *Quercus variabilis* | 32466 | 29808 | 2658 | 326 | 2693 |
| *Vitis vinifera* | 31315 | 27616 | 3699 | 814 | 3015 |
| *Xanthoceras sorbifolia* | 24672 | 23952 | 720 | 286 | 938 |
